# Social closeness modulates brain dynamics during trust anticipation

**DOI:** 10.1038/s41598-022-20827-y

**Published:** 2022-09-29

**Authors:** Said Jiménez, Roberto E. Mercadillo, Diego Angeles-Valdez, Juan J. Sánchez-Sosa, Jairo Muñoz-Delgado, Eduardo A. Garza-Villarreal

**Affiliations:** 1 Facultad de Psicología, Universidad Nacional Autónoma de México, Av. Universidad 3004, Copilco Universidad, 04510 Mexico City, CDMX Mexico; 2 Subdirección de Investigaciones Clínicas, Instituto Nacional de Psiquiatría Ramón de la Fuente Muñiz, Mexico City, Mexico; 3 Universidad Autónoma Metropolitana-Unidad Iztapalapa, CONACYT, Mexico City, Mexico; 4 Laboratorio de Cronobiología y Etología Humana, Instituto Nacional de Psiquiatría Ramón de la Fuente Muñiz, Mexico City, Mexico; 5 Laboratorio B-03, Instituto de Neurobiología, Universidad Nacional Autonoma de Mexico (UNAM) Campus Juriquilla, Boulevard Juriquilla 3001, Santiago de Querétaro, 76230 QRO México

**Keywords:** Psychology, Human behaviour, Cognitive neuroscience, Decision, Cooperation, Morality, Social behaviour

## Abstract

Anticipation of trust from someone with high social closeness is expected. However, if there is uncertainty in the interaction because a person is a stranger or because he has distrusted us on another occasion, we need to keep track of his behavior and intentions. Using functional Magnetic Resonance Imaging (fMRI) we wanted to find the brain regions related to trust anticipation from partners who differ in their level of social closeness. We designed an experiment in which 30 participants played an adapted trust game with three trustors: A computer, a stranger, and a real friend. We covertly manipulated their decisions in the game, so they trusted 75% of the trials and distrusted in remaining trials. Using a psychophysiological interaction analysis, we found increases in functional coupling between the anterior insula (AIns) and intra parietal sulcus (IPS) during trust anticipation between a high versus low social closeness partner. Also, the right parietal cortex was coupled with the fusiform gyrus (FG) and the inferior/middle temporal gyrus during trust anticipation of a friend versus a stranger. These results suggest that brain regions involved in encoding the intentions of others are recruited during trust anticipation from a friend compared to a stranger.

## Introduction

The anticipation of trust from an individual towards a person with high social closeness (i.e., a close friend) could be considered the status quo of interactions between members of the same group^1,2^. From borrowing a friend’s pen to requesting him to endorse a bank loan, these are situations that reflect the trust that is anticipated between members of the same social network. The expectation of trust from a person who shares high social closeness with another, allows their behavior to be predicted very accurately and generates the preconditions for cooperative and reciprocal interactions to occur^2,3^. However, if the other person is a stranger (someone with low social closeness) or if the context could imply risk due to social uncertainty, it is appropriate to anticipate that trust may not occur by default. Thus, anyone anticipating the trust of a partner needs to adapt their expectations and prepare for possible deviations from the social norm of trust, depending on information such as social closeness^4^. The trust placed from one person to another is regarded as a type of social reward^5^. The anticipation of this affiliative behavior seems to involve the activation of brain regions such as the ventral striatum (VS), which has been implicated in reward processing and has shown significant differential activity when people trust in-group versus out-group members (i.e., friends versus strangers)^6^. During the anticipation of a reinforcer, the salience network^7,8^ (SN), anchored in the anterior cingulate cortex (ACC) and the anterior insula (AIns), seems to be recruited to orchestrate motivational and attentional processes. In the context of a potentially prosocial interaction, the SN may help detect compliance or violation of social norms and could guide the decision to respond with reciprocity or else, to update the initial belief regarding the social preferences of the other person^9^. Therefore, it might also require the involvement of the Inferior Parietal Cortex (IPC), which tends to perform advanced social cognitive functions, such as the ability to infer thoughts, beliefs, and behavioral dispositions from others^10^.

Economic games are one of the main tools to study prosocial behavior and the associated neural circuits. In particular, the trust game allows exploring the brain mechanisms that underlie both the trustor's ability to place trust in others and the trustee's decision to respond reciprocally^11^. Although there are numerous studies that have investigated brain circuits involved in the decisions of both roles (trustor and trustee), little is known regarding the influence of social closeness on trust anticipation of the trustee. Even though it is true that the effect of social inputs has been found mainly related to the activity of isolated brain regions during cooperation, competition or approval (e.g., such as the VS or medial prefrontal cortex (mPFC))^12–15^, the neural dynamics during trust anticipation from a partner with high social closeness (a friend) compared to one with low closeness (stranger), is currently an open question.

In this study, we aimed to explore the neural dynamics of the AIns, the ACC, the VS and the inferior parietal cortex (IPC) during trust anticipation from a friend compared to a stranger; we use the named structures as seed regions of interest to conduct psychophysiological interaction (PPI) analyses^16,17^. We also wanted to evaluate whether the response of brain regions involved in anticipation is modulated by the partner's social closeness using whole-brain analysis. We were particularly interested in the activity of the right AIns and the ACC, due to the roles that the two areas play in the expectation of possible social norm violations, and their direct influence on regions that coordinate the executive and affective processes that underlie reciprocity or defection^18,19^. Also it has been proposed that the ACC detects the occurrence of a violation of social norms and the AIns generates an aversive experience that helps to calibrate the severity of the harm^4^. The VS is of interest because of its ability to activate differentially during the expectation of both primary and social reinforcers^6–8^. And the IPC because activation has been reported in tasks involving anticipation or prediction of prosocial behavior^20,21^, as well as in the processing of physiological and psychosocial stressors^22^. For these purposes, we manipulated social closeness in a trust game by introducing three partners: a computer (non-social control), a stranger (low social closeness), and a real friend considered close (high social closeness). In the trustee's role, the subjects independently anticipated the trust from their partners, and subsequently decided whether to reciprocate or not. While in the trustor's role, the partners decided whether to invest a monetary amount in the trustee, which could then result in higher profits for both. Given the social norm of trust^23^, we assumed that participants would anticipate investment from their trustors in most trials, especially if the trustor was their friend. Therefore, to ensure that our participants experienced the violation of the social norm and the associated emotional uncertainty, we covertly manipulated the behavior of the three trustors so that they randomly decided to distrust the participant in some of the trials.

Due to the AIns and ACC roles in the interoceptive signals representation and their belonging to the salience network^24^, our hypothesis was that, given the uncertainty regarding the behavior of their partners, the subjects will experience a more aversive sensation playing with a trustor of high social closeness than with one of low social closeness, which will be reflected in an increase in the AIns and ACC bold signals. Likewise, presumably due to the need to update the initial beliefs about the social preferences of their partners, we hypothesized that the AIns and ACC will be functionally coupled with limbic cortices and with mentalizing regions such as angular gyrus and posterior cingulate cortex in the default mode network^9^. According to the neuropsychological framework of third-party punishment, the neural dynamics would allow the individual to provide an estimate of the severity of the latent damage caused by a possible transgression, as well as infer the trustor's motivations^4^.

**Figure 1:**
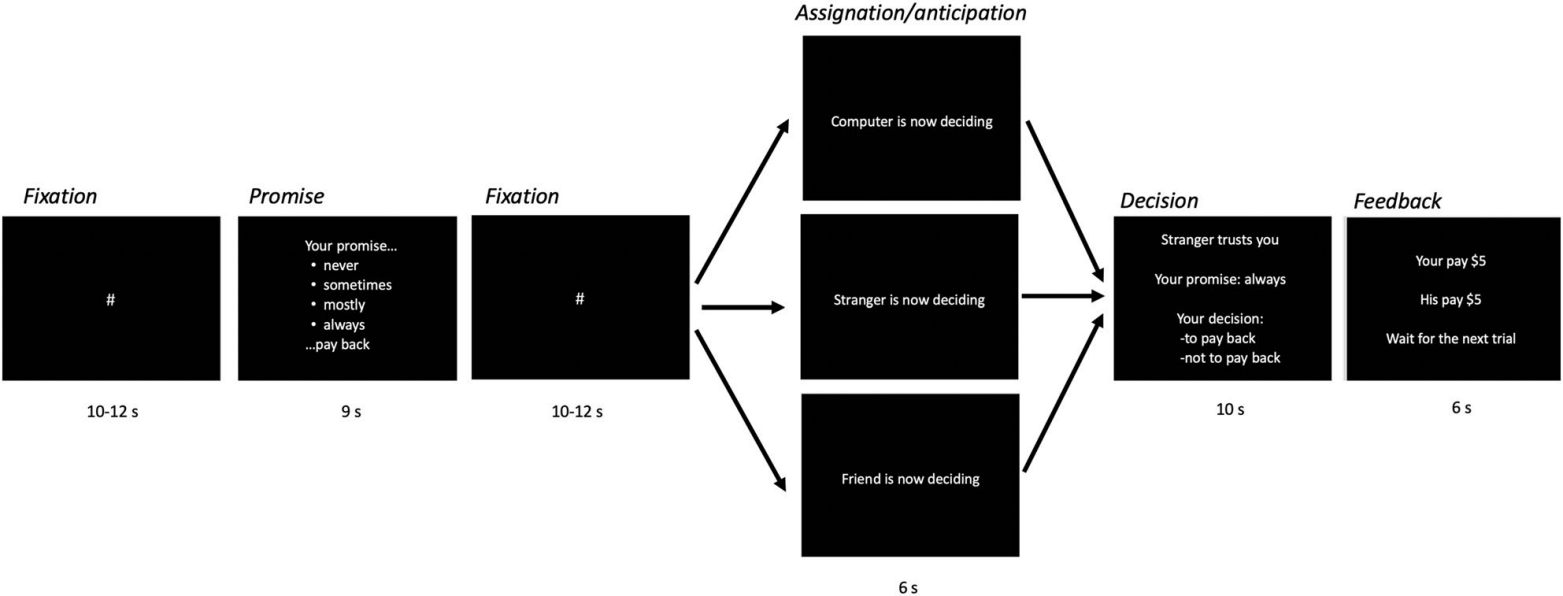
Task structure and timeline. From left to right the order and duration of the phases of a typical trial are shown; first, a fixation point was shown, then participants could make a promise regarding their payment frequency (Promise phase). After another fixation point, the subjects waited for 6 s their investor's decision, who could be the computer, the stranger, or the friend (Anticipation phase). In order for the participants to experience distrust of the investor, the decisions of the three trustors were programmed to randomly invest in 6 out of 8 trials in the participant, and in 2 out of 8 not to invest. During the decision phase, the participants decided whether to pay or keep the money. In the feedback decision, the payments for the current trial were shown. If the participant reciprocated, payments were $5 and $5, while if he did not, the payments were $10 and $0. In half of the trials, during the promise phase, a 9-s message was displayed indicating that it was possible to play without a promise, which provided the opportunity to contrast the participants' decisions when they made a promise and when they did not. Also, during 25% of the trials the trustors decided not to invest, in this case, during the decision phase, a message was shown saying that the partner had not invested, and in the feedback phase, it was indicated that the payments were $2 for the partner and $0 for the participant (Supplementary Table 1).

Another element included in the economic game was that the participants could make promises to their partners (Fig. 1). Promises were made to express the level of commitment to reciprocate that trustees had to trustors. In terms of the participants' behavior, the presence of promises was expected to increase the level of reciprocity compared to their absence^25^. Differences in the activity of the ACC and the AIns during the promise compared to the absence of the promise were also expected. According to other studies, this brain activity could reveal the potential intention (e.g. decision to keep the promise) of the one who makes the promise^20^. An interaction between promises and social closeness was also expected, specifically, it was hypothesized that the effect of promise on reciprocity decisions would be more significant towards the friend than towards the other investors. In half of the trials, the participants made a promise to their partners and then freely decided whether or not to keep it. The promises expressed participants' commitment to their investors on a scale from “never” to “always” I will return half of the profits. The strength of the commitment expressed in the promise would allow evaluating consistency between promises and decisions (i.e., I promised “always” and then decided to reciprocate), as well as dishonest behavior (i.e., I promised “always” and then did not pay). The participants also played without promises an equal number of trials, which allowed for a comparison in behavior and brain activity when people decided without promises in contrast to when they decided with promises. It should be noted that although the promises could be positive (always and mostly) or negative (never), in order to simplify the analysis and focus on social closeness, which was the main manipulation in the task, we just compared decisions in trials with promises versus without promises. A more detailed analysis of the promises can be reviewed elsewhere^26^.

## Results

### Social closeness

To ensure the level of social closeness that the participants and their friends reported, the two responded to the IOS scale “Inclusion of the Other in Self”^27^ without observing their friend's responses. It consists of seven pairs of circles that vary in the degree of overlapping and represent the subjective social closeness that one individual perceives with respect to another. Highly overlapping circles suggest high social closeness, while distant circles indicate the opposite. The participants were asked to answer the IOS scale about the friends, the stranger, and the computer. To verify the assumption that the participants experienced a similar degree of social closeness to their friends, we related the responses that the participant and their friends gave to the IOS scale. We found a positive significant correlation between the subject and friend subjective levels of social closeness (r = 0.44, 95% CI: 0.09–0.69, p < 0.05). A repeated measures ANOVA was also performed to assess whether closeness differs between the three game players. For the participant, a significant effect of social closeness on IOS scale response was found, F(2, 56) = 104.9, p < 0.001, Tukey's post hoc comparisons to determine differences between partners, revealed greater social closeness experienced towards the friend vs. the computer (i.e., higher IOS score), t(28) = 9.43, p < 0.001, as well as greater social closeness towards the friend compared to the stranger, t(28) = 17.53, p < 0.001. The Tukey post hoc comparison between the stranger and the computer was not statistically different, t(28) = 1.59, p = 0.27. For the friend, a significant effect of social closeness on the response to the IOS scale was also found, F(2, 56) = 265.77, p < 0.001. Therefore, social closeness between the MRI participants and their friends was met.

### Behavioral results

**Figure 2:**
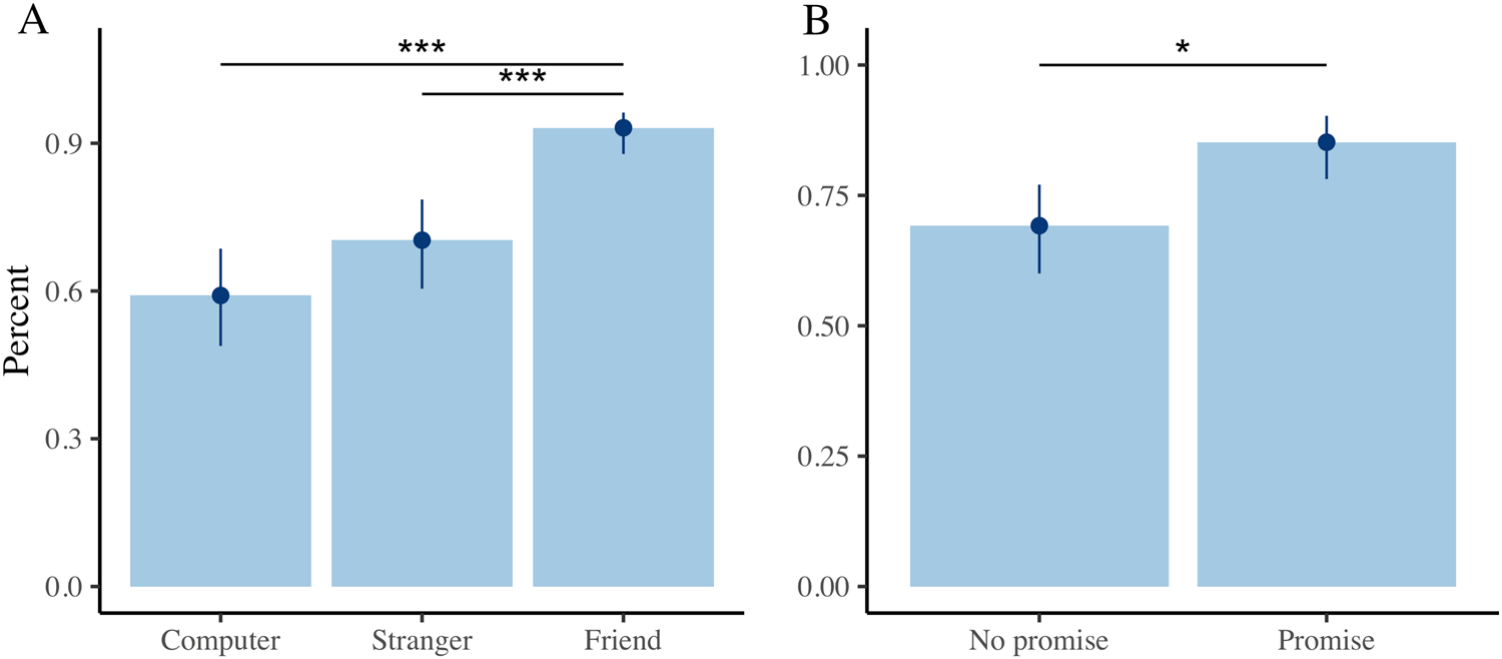
Effects of social closeness on reciprocity expressed by the decision to pay back in the Trust Game. (**A**) Percentage of payments depending on the level of social closeness of the trustor, the friend was paid significantly more than the stranger and the computer. The percentages of payment to the computer, stranger and friend were 59%, 70% and 93%, respectively. (**B**) Percentage of times that MRI participants decided to pay back when they made a promise compared to when they did not. The payment percentages in the trials with promises were 85%, while in the trials without promises they were 69%. *p < 0.05, ***p < 0.001.

Effects of promises and social closeness on the decision to reciprocate with trustors (pay back) were evaluated using a multilevel model with a binomial error distribution^28^. In the Trust Game, the participants had high levels of reciprocity as they decided to pay back to the trustors in 72% of trials regardless of social closeness and promises. However, the multilevel model using the Likelihood Ratio Test showed a significant effect of promises *X*^2^ (1, 30) = 15.23, p < 0.001, and social closeness *X*^2^ (2, 30) = 62.99, p < 0.001 on the decision to reciprocate. There were no significant interaction effects between promises and social closeness *X*^2^ (2, 30) = 1.06, p = . 59. Tukey post hoc comparisons showed significant effects of social closeness in reciprocation: the percentage that participants decided to pay back was statistically higher for the friend than the computer, Z = 5.36, p < 0.001, and stranger Z = 4.75, p < 0.001 (Fig. 2A). A significant increase in the decision to pay back was also found when participants made a promise compared to when they did not (Z = 2.22, p = 0.02) (Fig. 2B).

### Psychophysiological interaction (PPI) results

**Figure 3:**
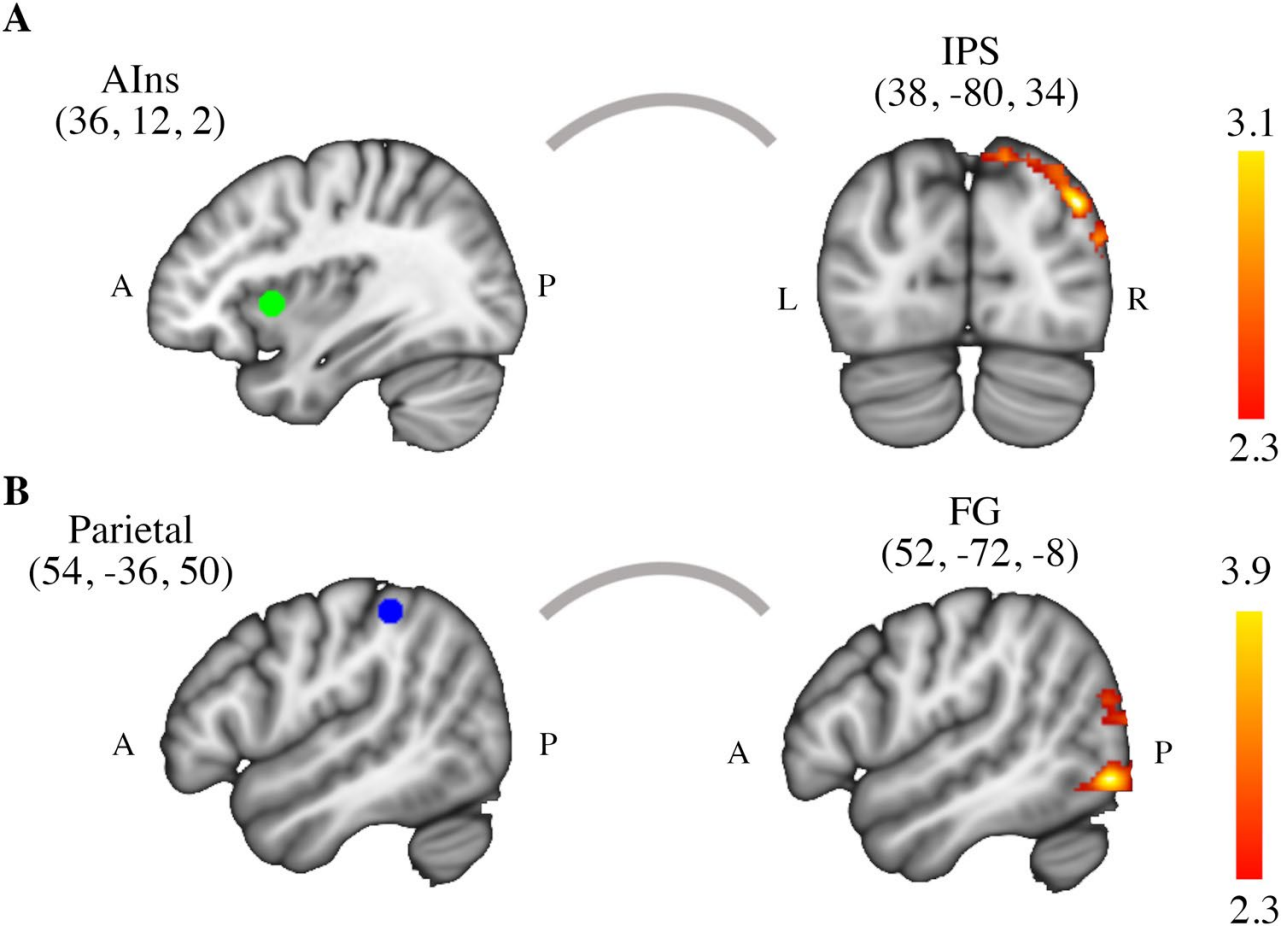
Psychophysiological interaction (PPI) results. (**A**) The right anterior insula (AIns) was the seed region (MNI coordinates 36, 12, 2). Greater functional connectivity was found between AIns and intra parietal sulcus (IPS) during anticipation of the friend's decision compared to the stranger. (**B**) The other seed was the right inferior parietal cortex (MNI coordinates 54, − 36, 50) which was coupled with the fusiform gyrus (FG) and the inferior/middle temporal gyrus during trust anticipation of a friend versus a stranger. Non-parametrically thresholded images using clusters determined by Z > 2.3 and a (corrected) cluster significance threshold of p = 0.05.

The investigation of brain dynamics with the PPI model revealed a significant interaction between the anticipation of a trustor's high vs. low social closeness and the time series of the right AIns. Specifically, during trust anticipation of the friend compared to the stranger, significantly functional coupling was found between the right AIns and the intra parietal sulcus (IPS) (peak Z-value = 3.14, p < 0.01, cluster-corrected). Likewise, the cluster of significant functional connectivity with the AIns, extended through the angular gyrus (AG), middle occipital gyrus, fusiform gyrus (FG), and middle temporal gyrus (Fig. 3A). Similarly, the right inferior parietal cortex (IPC) increased its functional connectivity with the fusiform gyrus (FG), and the inferior/middle temporal gyrus, during trust anticipation of a friend versus a stranger (peak Z-value = 3.98, p < 0.01, cluster-corrected) (Fig. 3B and Table 1). We did not detect any statistically significant activation cluster for PPI analyses with the ACC or VS as regions of interest.

**Table 1:** Psychophysiological interaction results (local maxima activations).

Contrast/seed region	Region	BA	MNI coordinates (mm)			Z-score	Cluster corrected p-value
			x	y	z		
Friend-Stranger	Middle occipital gyrus/Intra parietal sulcus	39	38	− 80	34	3.14	< 0.001
Anterior Insula (36, 12, 2)	Middle temporal gyrus	19	52	− 72	22	3.05	0.001
	Fusiform gyrus	37	66	− 60	8	2.90	0.002
	Middle occipital gyrus	39	44	− 76	34	2.83	0.002
	Angular gyrus	39	38	− 76	42	2.81	0.002
	Angular gyrus	39	42	− 74	40	2.81	0.002
Friend-Stranger	Fusiform gyrus	37	52	− 72	− 8	3.98	< 0.001
Inferior parietal (54, − 36, 50)	Inferior temporal gyrus	21	60	− 42	− 10	3.36	< 0.001
	Middle temporal gyrus	37	66	− 62	8	3.24	< 0.001
	Fusiform gyrus	37	64	− 56	− 6	3.20	< 0.001
	Middle temporal gyrus	21	62	− 34	− 8	3.19	< 0.001
	Fusiform gyrus	37	62	− 60	10	3.19	< 0.001

### fMRI main effects

**Figure 4:**
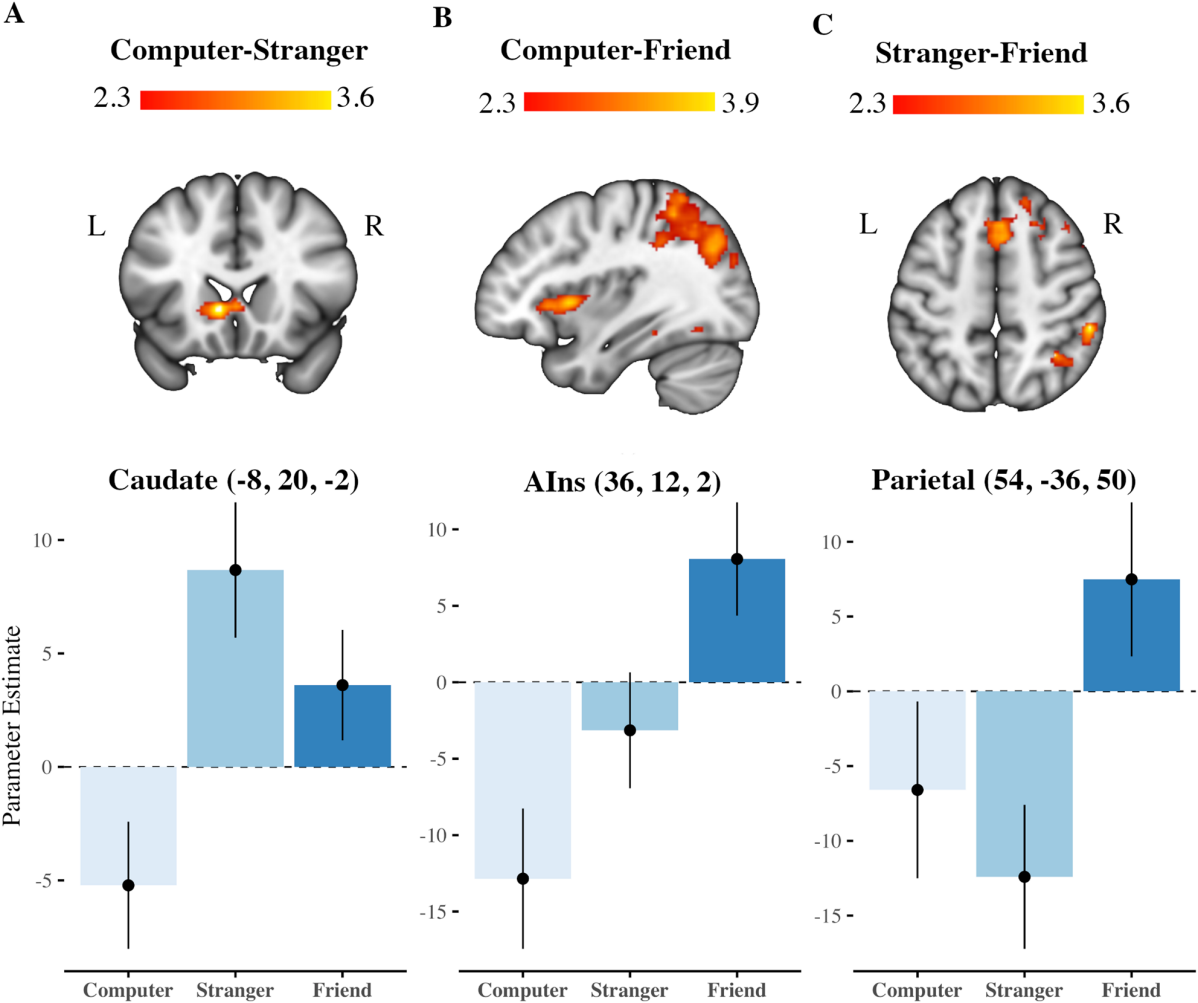
Neural regions involved in trust anticipation depending on the trustor’s social closeness. Whole-brain analysis regions involved during trust anticipation were detected with the 3 contrasts of interest. Top: (**A**), coronal view of the statistical map for the Computer—Stranger contrast, the voxels with higher activation during the trust anticipation phase are represented on an intensity scale between 2.3 < z < 3.6. (**B**), sagittal view of the statistical activation map for the Computer—Friend contrast, the voxels with the highest activation (highest Z-score) during the anticipation phase of trust are represented on an intensity scale between 2.3 < z < 3.9. (**C**), axial view of the activation statistical map for the Stranger-Friend contrast during the trust anticipation phase, plotted on an intensity scale between 2.3 < z < 3.6. Bottom: This shows the value of the parameter estimated at the peak of activation depending on the social closeness.

The whole-brain analysis identified the brain regions that showed significant activation during the anticipation of the trustor's decisions, the contrast Computer-Stranger recruited activation of the basal ganglia: caudate nucleus (peak Z-value = 3.68, p < 0.05, cluster-corrected) and putamen (peak Z-value = 3.63, p < 0.05, cluster-corrected) (Fig. 4A). The difference in activity during anticipation of the friend's decision compared to the computer’s decision, expressed by the contrast Computer-Friend, showed the maximum activation peak in the lingual gyrus (peak Z-value = 4.00, p < 0.05, cluster-corrected), also it revealed significantly activated clusters that included the angular gyrus, cuneus, precuneus, putamen and the AIns (Fig. 4B). Finally, the contrast Stranger-Friend found activation in the supplementary motor area (SMA) (peak Z-value = 4.00, p < 0.05, cluster-corrected), the middle frontal gyrus (MFG), the parietal lobe, and the angular gyrus (Fig. 4C). Coordinates of the peak activations for all contrasts are shown in Table 2. In a second GLM performed as sensitivity analysis, we did not find any statistically significant activation cluster for the contrast Promises vs. No promises, nor Pay back vs. No pay back. However, the results of the contrast of interest (Computer vs. Stranger, Computer vs. Friend, and Stranger vs. Friend) remained the same as those mentioned above.

**Table 2:** Whole-brain local maxima activations.

Contrast	Region	BA	MNI coordinates (mm)			Z-score	Cluster corrected p-value
			x	y	z		
Stranger > Computer	Caudate	48	− 8	20	− 2	3.68	< 0.001
	Putamen	49	− 20	8	− 12	3.63	< 0.001
	Caudate	48	0	16	0	3.56	< 0.001
	Putamen	49	− 32	0	0	3.08	0.001
	Putamen	49	− 24	2	4	2.97	0.001
	Putamen	49	− 18	12	2	2.97	0.001
Friend > Computer	Angular gyrus	7	28	− 50	44	3.88	< 0.001
	Lingual gyrus	19	− 20	− 54	− 10	4.00	< 0.001
	Cuneus	18	− 12	− 86	26	3.44	< 0.001
	Precuneus	7	− 12	− 62	66	3.41	< 0.001
	Anterior Insula	13	36	12	2	3.65	< 0.001
	Putamen	49	− 32	− 8	− 4	3.39	< 0.001
Friend > Stranger	Supplementary motor area	6	6	22	64	3.60	< 0.001
	Supplementary motor area	8	4	14	48	3.45	< 0.001
	Supplementary motor area	8	2	22	48	3.38	< 0.001
	Middle frontal gyrus	8	42	22	46	3.32	< 0.001
	Supplementary motor area	6	16	14	64	3.28	< 0.001
	Middle frontal gyrus	8	26	24	44	3.25	< 0.001
	Inferior parietal	40	54	− 36	50	3.60	< 0.001
	Inferior parietal	7	34	− 52	50	3.60	< 0.001
	Middle occipital gyrus	7	32	− 72	40	3.46	< 0.001
	Angular gyrus	39	46	− 58	44	3.34	< 0.001
	Inferior parietal	39	36	− 50	42	3.10	< 0.001
	Superior parietal	39	40	− 58	58	3.08	0.001

## Discussion

This study aimed to investigate the brain dynamics related to trust anticipation and social closeness. We found that subjects in the trust game decided to pay back more to friends than to a stranger and the computer. When anticipating friend’s decision vs. the stranger’s, using the psychophysiological interaction (PPI) analysis we found both higher functional connectivity between the right anterior insula (AIns) and the intra parietal sulcus (IPS); and interaction between inferior parietal cortex and fusiform gyrus (FG). During the anticipation of the stranger’s decision, using whole-brain analysis we found higher activity in the caudate nucleus and putamen than the computer’s decision, while there was higher activity in the lingual gyrus, angular gyrus, cuneus, precuneus, putamen and anterior insula in the anticipation of the friend’s decision vs the computer. We also found higher activity in the supplementary motor area, middle frontal gyrus, parietal lobe and angular gyrus when anticipating the friend’s decision vs. the Stranger’s. We did not detect statistically significant interaction of the anterior cingulate cortex (ACC) or ventral striatum (VS), as seed regions of interest. Our results suggest that the AIns is sensitive to trust anticipation from a human partner, regardless of their social closeness, and that AIns and IPC interact differentially with the middle occipital and temporal gyrus, as well as the IPS, depending on the social closeness of the trustor.

We propose that the activation pattern revealed by the friend vs. computer contrast, particularly the activity of the AIns, can be interpreted as the experience of internal conflict in the face of uncertainty regarding the decision of the closest partner. In a similar study, in which participants played the dictator game with close partners who varied in the relationship valence (friends vs. dislike partners), the authors found that people who were less prosocial toward their friends compared to their dislike partners, had greater activation of the supplementary motor area (SMA) and AIns during the game^29^. In this study, acting prosocial towards a friend is interpreted as a social norm, and not acting according to it induces internal conflict. We consider that the internal conflict could indicate both, acting against a social norm and norms deviation anticipation by other agents. Studies that indicate that being treated unfairly coincides with the AIns activity could support this idea^30,31^. Likewise, we propose that the AIns activity that occurs even before the subjects can act reciprocally, is sensitive to social information. It is very highly possible that these social inputs, such as the partner's closeness or the uncertainty regarding their behavior, have a significant impact on the process of assigning values to alternatives, that underlie reciprocal or selfish decision-making, and that it is probably occurring during the anticipation phase^32,33^. Thus, the greater AIns activity during the anticipation of a human trustor it is possible explained through internal conflict, caused by the possibility of being treated unfairly by the partner with the greatest social closeness. It is essential to add that the AIns could perform computations related to both the anticipation of cooperation, as well as deviations from the norms^2,18,19^, therefore its activation during the anticipation phase is consistent with both possibilities. It should be noted that despite the possible internal conflict, we found that the greater the trustor's social closeness, the greater were the reciprocal decisions of our subjects. Although this result could seem contrary to the internal conflict hypothesis, the neural dynamics between AIns and IPS, as well as their role in regulating the aversive experience through analysis of the underlying motivations, could explain these behavioral results^21^.

The greater activation of the fusiform gyrus (FG) in trust anticipation from friends compared to strangers is an interesting result. The FG, which underlies our ability to process faces to interact in a socially appropriate way, has also been involved in the anticipation of monetary rewards^34^, and has been found to increase their response to emotional stimuli with low and high social complexity^35^. It has been suggested that there is a neural network that includes the posterior FG and the inferior occipital gyrus, which specializes in identifying visual signals of high emotional importance. In addition, it has been proposed that the functioning of this network is fundamental during empathic reactions underlying a social interaction^35^, and that its alterations, could partially explain the social dysfunction observed in patients with autism spectrum disorders^36^. Thus, we speculate that the differential activation of the FG during the anticipation of the high vs low closeness partner could suggest the occurrence of an attentional process, which, maybe motivated by the uncertainty regarding the partners' decisions, is probably more demanding during the anticipation of the friend compared to the stranger. Attention during anticipation would be essential when monitoring the decisions of the friend partner because receiving their trust (distrust) could be perceived as more rewarding (punishing) than receiving the decision consequences of a stranger. However, the previous interpretation is definitely a conjecture, future research that uses experimental tasks with social nature could use the FG as a seed region in psychophysiological interaction (PPI) models, or in other region of interest (ROI) based analysis.

Using a PPI approach we detected greater functional connectivity between the right AIns and the middle temporal sulcus, during decision's anticipation from a friend versus a stranger trustor. Likewise, during the mentioned psychological context, the right AIns exhibited greater interaction with the IPS and the superior division of the lateral occipital cortex. These results suggest that: (1) The AIns is sensitive to trust anticipation from a human partner, regardless of their social closeness, presumably to encode aversive states caused by potential deviations from the trust social norm^30,31^, and (2) the AIns interacts differentially, depending on the social closeness, especially with the middle temporal gyrus and IPS, possibly to regulate the aversive experience by analyzing the intentions and objectives underlying the partner's trust^35^.

The neural dynamics observed in our study may be understood through the Punishment Neuropsychological Framework (PNF)^4^. Although punishment was not directly evaluated in the present work, our experiment allowed us to assess the anticipation of behaviors that might warrant punishment. The PNF proposes that humans frequently punish others whose behavior deviates from the norms, e.g., direct victims of violations can retaliate against their aggressors (second party punishment), even people who were not directly harmed are willing to punish who transgresses (third party punishment)^37^. How willing people are to punish depends on the harm that was done and the intentions of the offender^38^. Brain regions belonging to the salience network (SN) and the default mode network (DMN) are thought to be involved in the detection of deviations from social norms, also encoding of the damage's severity, and the intention assessment^4,39^. In particular, it has been proposed that the AIns represents social norms and generates aversive experiences depending on whether there is a violation, or harm threat^2^, while the Temporal Parietal Junction (TPJ) and the temporal sulcus represent the nature of the social interaction, in terms of the intentions or objectives of the transgressor^40–43^ and even encode social distance^44,45^. Our whole-brain results are congruent with the AIns' role in the information encoding related to anticipation of deviations from social norms^46^, however, we also show that the AIns interacts with the temporal and parietal regions depending on the trustor's social closeness. The functional coupling between these regions that we observed during trust anticipation from a high vs low social closeness partner, could reflect the information flow between the SN and the DMN (particularly between the AIns, the TPJ, and the posterior superior temporal sulcus), necessary to modulate the differential negative affect, produced by the uncertainty regarding the friend's behavior compared to the stranger. It is reasonable to speculate that the effect of the social cognition network when monitoring the intention of a proximate behavior, decreases the response of SN's regions (e.g. the amygdala), in a similar way to high-level regulatory strategies, affect emotional experiences^38,47^. A related result to the previous idea was that the whole brain analysis detected ventral striatum (VS) activity during the anticipation of the human trustor's decisions relative to the non-social control, however, this region was absent in our contrast to evaluate the difference between friend and stranger's anticipation. Also, the PPI analyses failed to detect any statistically significant interaction of the ACC and VS with other brain structures during the anticipation phase. In agreement with the PNF and the supposed modulation of affect, it is plausible that the VS and ACC responses could have been inhibited, yet, this hypothesis could be evaluated in future studies.

### Strengths and limitations

Our study has several strengths, beyond just focusing on whole brain activations during the anticipation of trustor’s decisions, the PPI model we used allowed us to evaluate the functional interaction of this region with other brain structures. PPI allows investigating not only individual regions involved in the task but also how is the information flow between brain areas and how functional regions change their connectivity in different psychological contexts^16,17^. The PPI analysis strengthens the study in terms of the sensitivity of our neuroimaging findings, while reducing the impact of the sample size, which could be considered relatively small for imaging studies. Another strength of the study was the participation of real-life partners (close friends), who although their decisions were programmed, the expectation of their presence during the game increases the validity of the task and results^29^. However, we also had important limitations that must be considered. The PPI does not allow establishing the directionality of the information flow between brain areas^48^, so we do not know if the AIns and parietal inferior receive or send information from the areas they interact with. Although we tried not to make inferences regarding the direction of the relationship between regions, our hypothesis that the mentalization network modulates the aversive experience in the face of the potential distrust of the friend could assume directionality of the information from the temporal areas to AIns. Therefore, future studies should empirically evaluate this question through effective connectivity methods such as Granger causality analysis or dynamical causal modeling^38^. Another limitation of the present study was the use of hypothetical monetary rewards rather than real, it could be argued that the subjects might not be sufficiently motivated by the consequences of decisions in the game. However, in the research literature on decision making, there are numerous studies that have explicitly addressed the difference between hypothetical versus real monetary rewards, without finding effects of the type of reward in self-control, temporal or social discount tasks^49,50^. Considering the aforementioned studies, as the findings of this work have theoretical congruence, there are few reasons to believe that other types of incentives would have led to different results.

Human social life success depends to a large extent on people trusting and taking risks together, with the purpose of achieving objectives that otherwise would fall short of reach. When it comes to interactions between members of the same group, the default is to anticipate the trust and reciprocity of close others like our friends. However, in societies as numerous and complex as human ones, it is frequent that many of our interactions occur with people hardly known or strangers. Although it may be less frequent, it is also possible that close group members prefer, in some circumstances, not to take the risk of placing their trust in us. In this way, anticipating the trust of another individual requires that we be able to selectively attend to socially relevant stimuli, such as closeness or other's past decisions, to generate adequate expectations regarding their behavior. And in case of anticipating a deviation from a social norm, analyze the motivations or objectives of the involved person, and make a motivated prosocial or proself decision. This complicated neuropsychological process requires the information flow between neural regions sensitive to social nature data, such as the AIns and IPS, which functionally interact to signal the other person closeness and modulate the aversive response that occurs as a consequence of potential distrust.

## Methods

### Participants

We recruited 30 healthy subjects (15 female), all reported being right-handed, and ranged between 19 and 33 years old (M = 23.7, SD = 3.71). Except for one participant who said she was married and studying for a postgraduate degree, all the other participants reported being single and undergraduate. No subject disclosed a neurological history or psychiatric illness. The participants attended our study with a “close” friend (n = 30), who had the following characteristics: (1) they were match-gender friends paired with the participant, (2) they were not a relative, and (3) they were not a romantic or sexual partner. Exclusion criteria were related to MRI safety such as claustrophobia or ferromagnetic metals in the body. The study was approved by the ethics committee of the Instituto Nacional de Psiquiatría Ramón de la Fuente Muñiz in Mexico City. All participants and their friends gave written consent for the study, and we followed the guidelines of the Declaration of Helsinki.

### Procedure

A researcher explained participants they would be scanned with MRI while playing an economic game (experimental fMRI task) with three partners: (1) their friend, (2) stranger (same sex) and (3) a computer. They were told that their friend was going to play with them in an isolated room, and that the stranger was another person who already knew the game and was waiting in another room for the moment, although they never met the stranger. We also told them that the computer partner was programmed to make decisions that benefit it. In reality, the participants were deceived as they did not play with anyone, and the responses and behavior of the friend, stranger, and computer were all programmed a priori to control the response variability. This deception was necessary as an experimental manipulation to ensure the effect of social closeness not to be affected by the real-time responses, to induce a level of distrust in the participant, as well as to control the timing of the study. To ensure the level of social closeness that the participants and their friends reported, the two responded to the IOS scale “Inclusion of the Other in Self”^27^ without observing their friend's responses. It consists of seven pairs of circles that vary in the degree of overlapping and represent the subjective social closeness that one individual perceives with respect to another. Highly overlapping circles suggest high social closeness, while distant circles indicate the opposite. The participants were asked to answer the IOS scale about the friends, the stranger, and the computer. After the verbal explanation of the economic game task, the participants were trained first outside the MRI scanner, then inside to get accustomed. Following training, the experiment began and lasted for 1 h. At the end of the experiment, the participants and their friends were told about the deception. All of our participants were given the opportunity to be eliminated from the study if they did not agree with any of the manipulations and deceptions performed by the investigator, however, none of our subjects chose that option.

### Experimental task

The task was programmed in PsychoPy 1.84.2^51^ and the participant observed the task on a viewer adapted for use inside the scanner, and responded by pressing two buttons on the response pad Lumina PAIR Pad of Cedrus, one of the buttons was used to the decision to “to pay back”, while the other was to “not to pay back”. The participants played the role of trustee in a trust game^52^(the task) against three trustors of different degrees of social closeness: computer (control), stranger (low), and friend (high). Each trial included 4 phases: (1) promises, (2) trust anticipation, (3) decision, and (4) feedback. During the promise phase, participants had to promise their partners how often they would reciprocate; during the trust anticipation phase, participants had to wait for their partners' decision about giving them money. In the decision phase, the participant had to decide whether or not to pay back to his trustor and, finally, during the feedback phase, payments for the trial were indicated depending on the decisions of the participants or the trustor's response (Fig. 1). The game consisted of 24 trials (8 for each partner) using hypothetical rewards: the trustor (computer, stranger, or friend) expressed his trust by investing $2 (Mexican pesos) in the trustee (participant), the trustee anticipated their partner's decision for 6 s; if the trustor trusted, the $2 would be multiplied by 5 and delivered to the trustee, while the trustor ran out of money. Later, if there was an investment, the trustee had to decide whether to return half to the trustor (trustee $5, trustor $5) or keep all the money (trustee $10, trustor $0). If there was no investment, the trustee received nothing in that trial and waited for her next partner (trustee $0, trustor $2). The three trustors (friend, stranger, and computer) were presented in a pseudorandom order and their decisions were programmed to randomly trust 6 out of 8 trials and distrust 2 out of 8. The order of all experimental conditions was the same for all participants (Supplementary Table 1).

### Image acquisition and preprocessing

Brain images were acquired using a Phillips Ingenia 3T MR system scanner (Philips Healthcare, Best, The Netherlands, and Boston, MA, USA), with a 32 channel dS Head coil. Functional data were acquired using a T2*-weighted echo-planar image sequence, with a repetition time (TR)/echo time (TE) = 20,000/30 ms, flip-angle = 75°, and inversion recovery for cerebrospinal fluid suppression. A total of 510 axial slices were acquired with an isotropic resolution of 3 mm, field of view = 240 mm, and acquisition matrix = 80 × 80. The structural data were acquired by means of a T1-weighted sequence with TR/TE = 7/3.5 ms, flip angle = 8°, field of view = 240 × 240, 1.0 mm isotropic voxels, acquisition plane = sagittal. MRI data were analyzed using FSL 6.0.1 (FMRIB’s Software Library). For preprocessing, each 4D volume was motion and slice timing corrected, and normalized onto MNI common brain space (Montreal Neurological Institute, EPI Template, voxel size 2 mm × 2 mm × 2 mm). Data were then smoothed using a Gaussian filter (full width half maximum = 6 mm) and highpass filtered with sigma = 50(s).

### Psychophysiological interaction (PPI) analyses

To investigate the neural dynamics during trust anticipation, four psychophysiological interaction (PPI) analyses were performed. We investigated if the functional connectivity of four ROIs of interest, the right AIns (MNI coordinates 36, 12, 2), the IPC (MNI coordinates 54, − 36, 50), the ACC (MNI coordinates − 6, 33, 6) and VS (MNI coordinates − 20, 10, − 8), was greater during the anticipation of a friend's decision compared to the anticipation of a stranger. Very close to these areas, activation has been reported in tasks involving anticipation or prediction of prosocial behavior^20,21^, as well as in the processing of physiological and psychosocial stressors^22^. The analyses were performed in FMRIB-FSL, using the temporal activation series of the mentioned brain regions, the activity was extracted from spherical ROIs of 5 mm radius, then the four PPI models were fitted with 7 regressors as a first-level analysis. The first explanatory variable was the task regressor (PSY) that included the anticipation phase for the stranger and friend's decisions, it had a duration of 6 s, a weight of − 1 was included for stranger's anticipation and 1 for friend's anticipation so that this regressor embodied the contrast Friend > Stranger; the second regressor was the physiological (PHY), for this, the right AIns, the IPC, the ACC and VS time-series activity during the entire task were used. The third regressor was the interaction between the psychological and physiological regressors (PSY*PHY). The remaining 4 regressors of the PPI model were covariates of no interest, three of them modeled the other task's phases, one for the promises phase (9 s), another for the control condition without promises (9 s), and another for the anticipation of the computer's decision (6 s). The 4th of the no-interest regressor was used to model the shared variance between the anticipation phase of the friend's decision and that of the stranger, it had a duration of 6 s and included a weight of 1 for the anticipation of the two investors. To identify group-level activations, we performed a one-sample t-test as a higher-level analysis. The normalized statistical images were thresholded non-parametrically using clusters determined by Z > 2.3 and a (corrected) cluster significance threshold of p = 0.05^53^.

### Whole-brain analysis

In order to examine the effect of social closeness on the BOLD signal of the brain regions involved in the trust anticipation, a first-level GLM was performed with 12 regressors, 6 movement regressors, 3 regressors of interest modeled the signal during the anticipation phase, and they lasted 6 s each. One regressor was included to model the anticipation of the computer's decision, a second regressor for the stranger, and a third regressor for the friend. Then, 3 regressors of no interest were included during the promises phase (9 s), during the decision phase (10 s), and during the promise phase control condition, which showed for 9 s the message that said: "you can play without promises". As part of the first-level analysis, three contrasts based on the hypothesis of interest were estimated: (1) the difference during the anticipation of the stranger investor compared to the computer (Stranger > Computer), (2) the difference during the anticipation of the friend investor compared to the computer (Friend > Computer), and (3) the difference during trust anticipation from a high compared to a low social closeness partner (Friend > Stranger). As sensitivity analysis and to assess brain regions activated during the promise phase and during pay back decisions, we specified a second first-level GLM whose decision phase was modeled with two regressors of 10 s each, the first regressor modeled the BOLD signal when the subjects made the decision to pay back and the second was when the subjects decided not to pay back. In this second GLM we specify the contrasts Promises > No promises and Pay back > No pay back. The other regressors and contrasts of the second first-level GLM were specified in the same way as the first. For statistical inference in both first-level GLMs, we performed a one-sample t-test for each contrast as a higher-level analysis, the normalized statistical images were thresholded using the same parameters referred to in the PPI analyses.

### Behavioral data analysis

Effects of promises and social closeness on the decision to reciprocate with trustors (pay back) were evaluated using a multilevel model with a binomial error distribution^28^, which was programmed in R with the *lme4* package^54^. To model the decision to pay back, the next categorical predictors were included as population-level effects (fixed effects): promises (with two levels: without promises/with promises), social closeness (with three levels: computer/stranger/friend), and the interaction promises by social closeness. The post hoc differences between trustors were analyzed using p-values adjusted with Tukey correction. The model also included the effect of social closeness at the individual level (random effects), to consider within-subject variability. The responses to the IOS scale were analyzed with a Pearson correlation to determine the association between the social closeness of the participant and their friend. Furthermore, to explore differences in experienced social closeness towards the different trustors, we computed two repeated measures ANOVA’s with the *afex* R package^55^, one of the analyses was for the participant's responses towards their partners (computer, stranger and friend) and a second analysis for the friend's responses to the participant, the stranger, and the computer.

## Supplementary Information

Supplementary Table S1.

## Data Availability

The datasets generated during and/or analyzed during the current study are available from the corresponding author on reasonable request.
