# Supplementary material for: Social closeness modulates brain dynamics during trust anticipation
Source: Sci Rep. 2022 Sep 29;12:16337. doi: 10.1038/s41598-022-20827-y (PMC9522769; doi:10.1038/s41598-022-20827-y)
Supplement: Supplementary file 1 — Supplementary Table S1. [file 41598_2022_20827_MOESM1_ESM.docx]

*Table S1. Full order of experimental conditions in the economic game*

| **Trial** | **Promise** | **Partner** | **Investment** |
| --- | --- | --- | --- |
| 1 | Promise | Stranger | Trust |
| 2 |  | Computer | Trust |
| 3 |  | Friend | Trust |
| 4 | No promise | Computer | Trust |
| 5 |  | Computer | Trust |
| 6 |  | Friend | Trust |
| 7 | Promise | Friend | Trust |
| 8 |  | Stranger | Distrust |
| 9 |  | Computer | Trust |
| 10 | Promise | Friend | Trust |
| 11 |  | Friend | Distrust |
| 12 |  | Stranger | Trust |
| 13 | No promise | Computer | Distrust |
| 14 |  | Friend | Trust |
| 15 |  | Computer | Trust |
| 16 | No promise | Stranger | Trust |
| 17 |  | Friend | Distrust |
| 18 |  | Stranger | Trust |
| 19 | Promise | Computer | Distrust |
| 20 |  | Stranger | Trust |
| 21 |  | Computer | Trust |
| 22 | No promise | Friend | Trust |
| 23 |  | Stranger | Distrust |
| 24 |  | Stranger | Trust |

Note. The experiment is balanced by promises and partners so that all participants experience those two conditions the same number of times. The decision to trust occurred 6 times for each partner and the decision to distrust occurred only 2 times. The objective of the above was to generate uncertainty in the participant regarding his partner's decision. The phase of promises or no promises only occurred during the indicated trials, in the two trials that appear with an empty cell in the table (immediately after the occurrence of the promise or no promise), the participants were reminded of what they had promised, or they were reminded to play without promises.
